# Correlates of metabolic syndrome among young Brazilian adolescents population

**DOI:** 10.1186/s12937-018-0371-9

**Published:** 2018-07-06

**Authors:** Michel Coutinho dos Santos, Ana Paula Cicci de Castro Coutinho, Mônica de Souza Dantas, Letícia Ayran Medina Yabunaka, Dartagnan Pinto Guedes, Silvia Aparecida Oesterreich

**Affiliations:** 1 University Hospital of the Federal University of Grande Dourados, Dourados, MS Brazil; 2 Northern University of Paraná, Londrina, PR Brazil; 3 Faculty of Health Sciences, Federal University of Grande Dourados, Dourados, MS Brazil

**Keywords:** Cardiometabolic risk, Metabolic syndrome, Lifestyle, Health promotion, Youth

## Abstract

**Background:**

Findings available in literature indicate that metabolic syndrome (*MetS*) diagnosed in young ages tends to remain in adulthood. The aim of the study was to identify demographic, nutritional, anthropometric and behavioral correlates of *MetS* in a sample of adolescents from Dourados, Mato Grosso do Sul, Brazil.

**Methodology:**

This is a cross-sectional school-based study involving 274 participants aged 12–18 years (186 girls and 88 boys). Anthropometric measurements were performed and a questionnaire with structured questions was applied for data collection. *MetS* was identified according to criteria proposed by the *International Diabetes Federation*. Data were statistically treated using bivariate analysis and hierarchical multiple regression.

**Results:**

The proportion of adolescents identified with *MetS* was equivalent to 4.7% [95% CI (3.6–6.0)]. Multivariate analysis showed that older age (OR = 1.22 [1.04–1.73]) and higher economic class (OR = 1.25 [1.07–1.96]) were significantly associated with *MetS*. Among behavioral factors, longer recreational screen time (OR = 1.26 [1.05–1.94]) and low fruits/vegetables intake (OR = 1.49 [1.23–2.41]) were independently associated with *MetS*. Likewise, excess body weight (OR = 1.52 [1.24–2.41]) was significantly associated with the outcome.

**Conclusion:**

The high proportion of adolescents with *MetS* and the identification of their correlates reinforce the need for early life style intervention and awareness programs in this population group.

## Background

Currently, regardless of sex, age, economic class and geographic region, overweight and obesity have become a global epidemic [1], which decisively contributes to the onset and development of metabolic syndrome (*MetS*) [2]. Findings available in literature indicate that *MetS* diagnosed in childhood and adolescence tends to remain in adulthood [3]. Therefore, the diagnosis of the possible presence of *MetS* at early ages, accompanied by control interventions, should have a favorable impact on the health of young people and the prevention of adverse outcomes in the future.

*MetS* refers to a set of cardiometabolic components that, when altered, favor the occurrence of cardiovascular events and diabetes. These components include excess abdominal fat, high blood pressure, and altered triglyceride, lipoprotein, and blood glucose levels [4]. Currently, for the adult population, there is consensus regarding the cutoff points used to define the *MetS* components. However, it is not the case of adolescents, where the diagnostic criterion varies considerably among the different available proposals, with repercussion in clinical practice, making it difficult the comparison among studies [5].

Even considering this limitation and due to the importance and the need to diagnose the presence of *MetS* and correlates as early as possible, a number of studies have been carried out involving adolescents [6–12]. This is necessary because the adult prevalence is on the rise [13], and in the young population, evidence has pointed to similar phenomenon [9–11]. In addition, several attributes linked to health risk behaviors have been increasingly frequent in adolescents [14].

Therefore, the aim of the present study was to identify demographic, nutritional, anthropometric and behavioral correlates of *MetS* in a sample of adolescents from Dourados, Mato Grosso do Sul, Brazil.

## Methods

In order to carry out the study, information from the database of a larger project with longitudinal characteristics (*Health Education Program through Dietary and Physical Activity Interventions*) was used, which includes adolescents enrolled in the 2nd cycle of elementary school (6th to 9th grades) and high-school (from 1st to 3rd grades) of four public schools randomly selected in the city of Dourados, Mato Grosso do Sul, Brazil. In this case, data were collected at the initial moment of the project. The intervention protocols were approved by the Research Ethics Committee of the Federal University of Grande Dourados (Protocol No. 1.434.947).

The inclusion of adolescents in the study was due to their desire to participate in the experiment. To that end, all students enrolled in the 2016 school year of the four selected schools, along with their parents/guardians, were contacted and informed of the nature and aims of the study. The criteria adopted to exclude some adolescents interested in participating in the study were: (a) any health problem that temporarily or permanently prevented participation in the study; (b) using any type of medication that could induce changes in the study variables; (c) undergoing any type of specific diet; and (d) pregnancy. Of the 1200 students contacted, 274 adolescents (186 girls and 88 boys) aged 12–18 years confirmed participation in the project and signed the Free and Informed Consent Form.

The study performed anthropometric measures and components related to the identification of *MetS*, and a questionnaire consisting of items distributed in four sections: demographic aspects, eating habits, physical activity and sedentary behavior, was applied. The questionnaire was applied in a single moment, individually for each adolescent and in the place and time of classes. Data were collected between June and July/2016 and were carried out by a team of four researchers.

Regarding demographic aspects, in addition to sex and age, information related to ethnicity, economic class, schooling of parents/guardians, family structure and eventual work activity were collected. The family economic class was identified according to guidelines proposed by the National Association of Research Companies [15]. Information related to eating habits was obtained using semi-quantitative food frequency questionnaire from the Youth Risk Behavior Survey (YRBS) module, which was translated, adapted and validated for use in the Brazilian young population [16]. In this case, adolescents answered how often they consumed fruits/vegetables and sweetened products/soft drinks, taking as reference the week before data collection. From the intake frequency reported, the following indicators were considered: no intake; intake 1–4 days/week; intake ≥5 days/week.

For the physical activity, the Physical Activity Questionnaire for Adolescents - PAQ-A was used, which was translated and validated for use in young Brazilians [17]. The PAQ-A consists of eight structured questions aimed at sizing different aspects of physical activity in the last seven days. The response options are coded using an increasing scale from 1 to 5 points, and the physical activity score was computed using the mean of scores assigned to each question. For the categorization of physical activity scores, specific cutoff points for sex and age based on the distribution of tertiles was used. Thus, the group of less active adolescents was stratified with PAQ-A scores ≤1st tertile. The group of moderately active adolescents had scores between the 1st and 2nd tertiles, and the group of the most active adolescents had scores ≥2nd tertile.

Sedentary behavior was evaluated by exposure to excessive screen time through structured issues about watching TV and using computer, video game, tablet, and smartphone in a typical week. A predefined time scale was provided for response, in which the adolescent indicated his option among four categories, ranging from “none” to “> 4 hours/day”. The questions considered separately screen time equivalent to watching TV and using computer, video game, tablet and smartphone on weekdays and on weekends (Saturday and Sunday). Weighted average involving data of weekdays and weekends was used to identify screen time per day reported by students. Excessive screen time was defined by the combined use of TV and other screen devices for time > 2 h/day [18].

In the anthropometric field, height, body weight and waist circumference measurements were performed according to methodology described by the World Health Organization [19]. Body mass index (BMI) was calculated using the ratio between body mass expressed in kilograms and height expressed in meters squared (kg/m^2^). With BMI values, the anthropometric nutritional status of students was classified into four categories based on sex and age cutoff points proposed by the International Obesity Task Force (IOFT): low body weight, eutrophic, overweight and obese [20].

*MetS* was identified by analyzing the blood content of plasma lipids (triglycerides and HDL-C) and blood glucose, resting blood pressure (systolic and diastolic) and abdominal fat accumulation (waist circumference), according to criteria proposed by the International Diabetes Federation [21]. In this case, *MetS* was defined by the presence of high waist circumference (< 16 years: both sexes ≥ Percentile 90, ≥ 16 years: boys ≥90 cm and girls ≥80 cm) and at least two other compromised components: increased triglycerides (≥ 150 mg/dL), low HDL-C (< 16 years: both sexes < 40 mg/dL, ≥ 16 years: boys < 40 mg/dL and girls < 50 mg/dL), high fasting blood glucose (≥100 mg/dL) and altered blood pressure (systolic ≥130 mmHg or diastolic ≥85 mmHg).

Data were statistically treated using the Statistical Package for Social Science (SPSS), version 22. The observed proportions (%) in the outcome of Interest (*MetS*) according to demographic, anthropometric, behavioral and nutritional correlates were presented with respective 95% confidence intervals (95% CI). To analyze the linearity of associations between *MetS* and potential correlates, prevalence ratio calculations were used. Statistical differences among strata under investigation were treated by the chi-square test (χ^2^). In the sequence, correlates that indicated at least marginally significant associations (*p* ≤ 0.20) in the bivariate analysis were selected to be included in hierarchical multiple regression procedures. In this case, correlates were included in blocks, and demographic aspects (level one) were the first to be included the model, followed by the anthropometric nutritional indicator (level two) and, finally, the behavioral components were included (level three). All correlates that presented statistical significance remained in the multivariate model, *p* < 0.05.

## Results

Descriptive information characterizing the sample selected for the study is provided in Table 1. Approximately 1/3 of the sample is composed of boys (32.1%) and the highest concentration of adolescents is aged 12–15 years (58%). Most adolescents in the study were Caucasian (72.6%), live with parents (66.4%) and did not work (76.3%). Regarding the economic class and schooling of parents/guardians, the adolescents were proportionally distributed in the strata considered. In addition, 25.9% of adolescents were overweight (overweight + obesity), almost half were classified as minimally physically active (49.7%), and in each group of ten adolescents, seven reported remaining over 2 h in screen devices. Daily fruit/vegetable intake was reported by 27% of adolescents and 93.4% of them assumed to consume sweetened products/soft drinks at least once a week. Regarding the individual components of *MetS*, decreased HDL-cholesterol was predominant (25.2%), while increased triglyceride (6.6%) and high fasting blood glucose (5.1%) were the least prevalent.

**Table 1:** Descriptive information of the sample selected in the study

	n (%)
Demographic Indicators	
Sex	
Girls	186 (67.9)
Boys	88 (32.1)
Age	
12–15 years	159 (58.0)
16–18 years	115 (42.0)
Ethnicity	
Caucasian	199 (72.6)
Non-Caucasian	75 (27.4)
Economic class	
Class D-E (Low)	99 (36.1)
Class C	107 (39.1)
Class B-A (High)	68 (24.8)
Schooling of Parents/Guardians	
≤ 4 years	82 (29.9)
5–8 years	59 (21.5)
9–11 years	55 (20.1)
≥ 12 years	78 (28.5)
Family structure	
Father and mother	182 (66.4)
Separated Parents	64 (23.4)
Relatives	28 (10.2)
Labor Activity	
None	209 (76.3)
Eventual	20 (7.3)
≥ 20 h/week	45 (16.4)
Nutritional status Anthropometric	
Body mass index	
Low weight	18 (6.6)
Normal weight	185 (67.5)
Overweight	42 (15.3)
Obesity	29 (10.6)
Behavioral indicators	
Physical activity	
Less active	136 (49.7)
Moderately Active	76 (27.7)
More active	62 (22.6)
Screen Time	
≤ 2 h/day	66 (24.1)
> 2 h/day	208 (75.9)
Fruits/vegetables intake	
No intake	44 (16.1)
Intake 1–4 days/week	156 (56.9)
Intake ≥5 days/week	74 (27.0)
Sweetened products/soft drinks	
No intake	18 (6.6)
Intake 1–4 days/week	126 (46.0)
Intake ≥5 days/week	130 (47.4)
Metabolic Syndrome Components	
High Waist Circumference	42 (15.3)
Increased Triglyceride	18 (6.6)
Decreased HDL-cholesterol	69 (25.2)
Elevated fasting blood glucose	14 (5.1)
Altered Blood Pressure	24 (8.8)

The presence of *MetS* with stratification for demographic, nutritional status and behavioral correlates are presented in Table 2. Overall prevalence was equivalent to 4.7% [95% CI (3.6–6.0)]. Results from bivariate analyses showed that, from the list of potential correlates considered, sex, ethnicity, family structure and labor activity were not statistically indicated (*p* < 0.20).

**Table 2:** Prevalence (95% CI) and prevalence ratio (95% CI) of metabolic syndrome with stratification for demographic, nutritional and behavioral correlates of adolescents from Dourados, Mato Grosso do Sul, Brazil, 2016

		Prevalence (95% CI)	Prevalence Ratio	*p*-value
	Overall	4.7 (3.6–6.0)		
Demographic Indicators				
Sex				0.217
	Girls	4.4 (3.5–5.4)	Reference	
	Boys	5.0 (3.8–6.4)	1.10 (0.95–1.41)	
Age				0.173
	12–15 years	4.2 (3.2–5.3)	Reference	
	16–18 years	5.2 (4.0–6.6)	1.21 (1.03–1.63)	
Ethnicity				0.341
	Caucasian	4.9 (3.8–6.1)	1.04 (0.92–1.29)	
	Non-Caucasian	4.6 (3.5–5.8)	Reference	
Economic class				0.139
	Class D-E (Low)	4.3 (3.3–5.4)	Reference	
	Class C	4.6 (3.6–5.8)	1.05 (0.93–1.39)	
	Class B-A (High)	5.4 (4.2–6.8)	1.23 (1.04–1.68)	
Schooling of Parents/Guardians				0.166
	≤ 4 years	4.2 (3.3–5.1)	Reference	
	5–8 years	4.6 (3.5–5.9)	1.07 (0.94–1.34)	
	9–11 years	4.9 (3.7–6.4)	1.14 (0.98–1.46)	
	≥ 12 years	5.2 (3.9–6.7)	1.21 (1.01–1.67)	
Family structure				0.367
	Father and mother	4.9 (3.8–6.1)	1.04 (0.93–1.30)	
	Separated Parents	4.6 (3.5–5.8)	Reference	
	Relatives	4.7 (3.7–5.8)	1.00 (0.89–1.36)	
Labor Activity				0.274
	None	4.9 (3.8–6.1)	1.03 (0.94–1.30)	
	Eventual	4.6 (3.4–5.9)	Reference	
	≥ 20 h/week	5.1 (3.9–6.5)	1.08 (0.95–1.35)	
Nutritional Anthropometric status				
Body mass index				< 0.001
	Low weight	4.0 (3.4–4.7)	Reference	
	Normal weight	4.4 (3.5–5.5)	1.08 (0.96–1.33)	
	Overweight	4.9 (3.8–6.1)	1.21 (1.02–1.69)	
	Obesity	5.6 (4.4–6.8)	1.38 (1.15–1.90)	
Behavioral indicators				
Physical activity				0.181
	Less active	5.1 (4.0–6.3)	1.19 (1.01–1.67)	
	Moderately Active	4.8 (3.8–5.9)	1.12 (0.96–1.45)	
	More active	4.2 (3.4–5.2)	Reference	
Screen Time				0.119
	≤ 2 h/day	4.2 (3.3–5.3)	Reference	
	> 2 h/day	5.3 (4.1–6.7)	1.24 (1.02–1.69)	
Fruits/vegetables intake				0.002
	No consumption	5.4 (4.2–6.8)	1.35 (1.13–1.91)	
	Intake 1–4 days/week	4.9 (4.8–6.1)	1.22 (1.03–1.73)	
	Intake ≥5 days/week	3.9 (3.2–4.8)	Reference	
Sweetened products/soft drinks intake				0.159
	No intake	4.3 (3.3–5.4)	Reference	
	Intake 1–4 days/week	4.6 (3.5–5.9)	1.04 (0.94–1.34)	
	Intake ≥5 days/week	5.2 (4.0–6.5)	1.20 (1.01–1.69)	

Results of the hierarchical multiple regression are available in Table 3. In the case of demographic correlates, the final model pointed to significant associations between *MetS*, age and economic class. Likewise, the anthropometric correlate related to nutritional status (BMI) remained significantly associated with *MetS*. Among behavioral components, of the four correlates considered, two of them (screen time and fruits/vegetables intake) remained associated with the outcome.

**Table 3:** Hierarchical multiple logistic regression for demographic (level 1), nutritional (level 2) and behavioral (level 3) correlates of metabolic syndrome of adolescents from Dourados, Mato Grosso do Sul, Brazil, 2016

Correlates	OR _Crude_ (95% CI)^a^	OR _Adjusted_ (95% CI)^b^
Level 1 – Demographic Indicators		
Age		
12–15 years	Reference	Reference
16–18 years	1.26 (1.03–1.88)	1.22 (1.04–1.73)
Economic class		
Class D-E (Low)	Reference	Reference
Class C	1.17 (0.98–1.73)	1.15 (0.97–1.61)
Class B-A (High)	1.29 (1.03–2.13)	1.25 (1.07–1.96)
Schooling of Parents/Guardians		
≤ 4 years	Reference	Reference
5–8 years	1.14 (0.95–1.77)	1.11 (0.93–1.71)
9–11 years	1.18 (0.97–1.95)	1.14 (0.96–1.82)
≥ 12 years	1.24 (1.01–2.06)	1.19 (0.98–1.89)
Level 2 – Nutritional Anthropometric status		
Body mass index		
Low weight	Reference	Reference
Normal weight	1.16 (0.97–1.77)	1.10 (0.95–1.69)
Overweight	1.25 (1.02–2.17)	1.18 (0.98–1.99)
Obesity	1.67 (1.29–2.78)	1.52 (1.24–2.41)
Level 3 – Behavioral indicators		
Physical activity		
Less active	1.23 (1.02–2.04)	1.18 (0.98–1.87)
Moderately Active	1.19 (0.99–1.83)	1.13 (0.96–1.69)
More active	Reference	Reference
Screen Time		
≤ 2 h/day	Reference	Reference
> 2 h/day	1.29 (1.05–2.11)	1.26 (1.05–1.94)
Fruits/vegetables intake		
No intake	1.59 (1.24–2.65)	1.49 (1.23–2.41)
Intake 1–4 days/week	1.29 (1.08–2.27)	1.24 (1.06–2.03)
Intake ≥5 days/week	Reference	Reference
Sweetened products/soft drinks intake		
No intake	Reference	Reference
Intake 1–4 days/week	1.15 (0.94–1.98)	1.09 (0.93–1.76)
Intake ≥5 days/week	1.21 (1.01–2.17)	1.14 (0.97–1.96)

^a^Unadjusted odds ratio

^b^Odds ratio adjusted by the other variables included in the model

## Discussion

The aim of the study was to provide up-to-date information on demographic, anthropometric and behavioral nutritional correlates of *MetS* in a sample of adolescents from Dourados, Mato Grosso do Sul, Brazil. The main findings were that, using the same diagnostic criteria (IDF), the proportion of adolescents with *MetS* was higher than that found in the young Brazilian population [4.5% vs. 2.6%] [12]. When compared with international data, the proportion observed in the present study is lower than that reported in North American and European adolescents; however, higher than that found in adolescents from Asian countries [9–11]. Also, in addition to being older and belonging to higher economic class, adolescents identified with *MetS* were those who reported excessive screen time, lower fruits/vegetables intake and greater body weight accumulation.

In general, correlates of *MetS* identified in the present study are consistent with current literature [6–8]. However, it is noteworthy that, unlike the findings of some studies [9, 10]; but consistent with others [11, 12], sex had no significant association with the presence of *MetS*. In this case, possibly the differences observed among studies can be attributed to the several criteria used to identify *MetS* in children and adolescents, since to date, there is no consensus regarding the use of a single criterion [5]. Another hypothesis to be considered may be related to the known differences between sexes related to the prevalence of abdominal obesity, hypertension and dyslipidemia found in different young populations [9]. in more advanced ages.

Corroborating previous findings [6–12], age was a significant correlate for the presence of *MetS* among adolescents in this study. In this regard, the expected increase in blood pressure, triglyceride and fasting blood glucose levels, and visceral fat deposits in more advanced ages [22] may explain the higher proportion of *MetS* among older adolescents. Regarding the economic class, the presence of *MetS* was positively associated with the higher strata. Other studies have identified significant associations in the same direction [6, 8]; however, we also find information in literature pointing to an inverse relationship between economic class and presence of MetS [12]. Perhaps the different indicators used to classify the economic class and the interactions between family income and attributes of the socio-cultural context in which it is inserted can contribute to understand these differences.

Regarding sedentary behavior, in a certain way, the results found here coincides with results found in other studies and suggest that the possibility of the presence of *MetS* is progressively higher according to the increase in screen time reported by adolescents [8, 23, 24]. However, a fact that should be considered refers to the cutoff point used to define excessive screen time, typically recommended by current international guidelines and used in the present study (> 2 h/day). In this sense, in a study involving meta-analysis resources, primary and sensitivity analyses performed based on this cutoff point did not reveal significant associations between screen time and *MetS*. In this case, individual studies included in the meta-analysis that indicated significant associations adopted cutoff points close to 4 h/day [25].

Regarding physical activity, no significant associations between scores equivalent to PAQ-A and *MetS* were found. To our knowledge, this was the first study that used PAQ-A to address possible associations between physical activity and *MetS*. However, according to current literature, it was verified that studies involving other types of questionnaires also found no significant differences in the physical activity of adolescents identified and not with *MetS* [26, 27]. On the other hand, studies that identified significant association between low levels of physical activity and greater chance of identifying *MetS* used the accelerometry technique [28] as a measure of physical activity. These findings are confirmed through meta-analysis resources, which demonstrated the reliance on the use of accelerometers to identify significant associations between physical activity practice and *MetS* in young populations [25].

Another finding from the present study was the significant association detected between fruits/vegetables intake and *MetS*. It is important to highlight that this food habit remained significantly associated even through adjustments to potential confounding variables. In this case, the protection attributed to higher fruits/vegetables intake is consistent with evidence presented by other studies involving different experimental designs and statistical treatments [6–8, 26]. Dietary pattern exerts influence on *MetS* through a specific effect on the plasma lipid-lipoprotein profile, blood pressure and body fat. Unlike diets in which fat-rich foods predominate, diets with higher fruits/vegetables intake tend to have lower intake of simple carbohydrates and saturated fat, while higher amounts of complex carbohydrates and fibers are inversely related to altered blood glucose and triglycerides, increased accumulation of abdominal fat and high blood pressure, and positively with more favorable HDL-C, all known components of *MetS* [29].

Based on the findings of the present study, the higher the excess body weight, the higher the proportion of *MetS*, with an abrupt increase in obesity cases. Similar findings are found in literature [6–9, 12], which corroborates the hypothesis that anthropometric nutritional status is strongly associated with *MetS*. In this sense, studies have detected important associations between obesity and *MetS* from early ages. Using a longitudinal design, among a set of biological and behavioral variables, it was found that childhood obesity is the strongest predictor of *MetS* and other risk factors for cardiovascular diseases in early adulthood [30].

The research method used to identify behavioral indicators involved a self-report questionnaire, thus allowing possible memory bias or even tendentious statements towards the desirable, being among the study limitations. However, the reporting of these indicators by adolescents themselves is a current procedure in studies with these characteristics, being the most feasible form of school-based or population-based surveys. In addition, the small sample size may somehow potentiate possible inaccuracy of the calculated estimates. The cross-sectional approach of data does not allow making inferences of causality in the association between the identification of *MetS* and the investigated correlates, because the outcome and the other variables have been identified at the same time.

## Conclusions

In conclusion, the results of the study are considered as a preliminary observation among the young Brazilian adults population as a high risk group for *MetS*. In approximately 5% of adolescents selected in this study the presence of *MetS* was identified, with emphasis on older individuals and those of higher economic class. Significant inverse associations between *MetS* and fruits/vegetables intake, coupled with the direct association with higher screen time and greater body weight accumulation, suggest that policies and interventions aimed at health education programs targeting school and family contexts should include actions focusing on the attempt to reduce the incidence of *MetS*.

## Abbreviations

BMI: Body mass index
HDLc: High density lipoproteins
IC95%: 95% Confidence intervals
IDF: International diabetes federation
IOFT: International obesity task force
MetS: Metabolic syndrome
PAQ-A: Physical activity questionnaire for adolescents
SPSS: Statistical package for the social science
YRBS: Youth risk behavior survey
χ2: Chi-square test
